# Meaning in great ape communication: summarising the debate

**DOI:** 10.1007/s10071-015-0936-3

**Published:** 2015-11-16

**Authors:** Thomas C. Scott-Phillips

**Affiliations:** Evolutionary Anthropology Research Group, Department of Anthropology, Durham University, Dawson Building, South Road, Durham, DH1 3LE UK

**Keywords:** Communication, Meaning, Intentionality, Language, Primates, Pragmatics

## Abstract

Does non-human great ape communication have meaning in the same way as human words (and some other human behaviours)? I recently argued that the answer to this question is most likely to be in the negative (Scott-Phillips in Anim Cogn 18(3):801–805, 2015a). Here, I (1) briefly respond to criticism of this view; (2) describe exactly what sort of empirical study could settle the matter; and (3) discuss what the best working hypotheses should be, in the absence of definitive empirical studies.

## Introduction

The question of meaning in non-human primate communication is important both for our understanding of ape minds in their own right, and for understanding the evolutionary origins of human communication (recent important contributions include Millikan 2004; Hurford 2007; Tomasello 2008; Rendall et al. 2009; Wharton 2009; Wheeler and Fischer 2012; Bar-On 2013; Stegmann 2013; Hobaiter and Byrne 2014; Scott-Phillips 2014). Adding to this literature, I recently published a short paper, in this journal, that described two different ways in which the term meaning can coherently be used in the context of communication, but only one of which corresponds to the way in which human words, and some other communicative acts, have meaning (Scott-Phillips 2015a). I also described what would need to be shown experimentally in order to conclude that a given behaviour is meaningful in this way. I argued that no case of non-human communication has yet been shown to satisfy this criterion, and hence concluded that there is at present no justified reason to assign meaning to the communication of non-human great apes, or any other non-human species. In a counter-article, also in this journal, Moore argues for the very opposite conclusion, namely that there are ample grounds to believe that great ape communication has meaning in the relevant sense of the term (Moore 2015). Here, I respond briefly to Moore’s arguments.

Let me begin my stressing one very important point of agreement. Give or take some matters of detail, there is consensus between Moore and myself, and indeed in much of the wider literature, that meaning involves overt intentionality, in which speakers produce signals with an intention that receivers recognise that the signaller has such an intention. I expand on this point in the next section. Debate begins not here, but with the subsequent question of how overt intentionality can and should be operationalised. Moore calls these “interpretational disputes” (p. 1). Expanding on points made in my previous paper, I here (1) describe exactly what experimental developments would advance discussion in the future, and (2) set out the options for what the best working hypotheses should be, based on our current state of knowledge.

## Meaning and overt intentionality

The vast majority of contemporary discussion of the notion of meaning has at its foundations the analysis of Paul Grice (1957; see also Grice 1969). Grice’s work has been adapted and built on in various ways since, in order to address a variety of concerns raised against it (Schiffer 1972; Neale 1992; Sperber and Wilson 1995; Recanati 2004). However, the Gricean insight that is most important for the present purposes is also the one that is most widely accepted. It is correspondingly retained, in one form or another, in most subsequent work. It is typically expressed as a pair of conditional clauses:

A speaker (or gesturer) *S* means something by an utterance *x* if and only if, for some hearer (or audience) *H*, *S* utters *x* intending: (1) *H* to produce a particular response *r*; and (2) *H* to recognise that *S* intends (1).

Clause 2 states that the speaker intends that the hearer recognises that the speaker has an intention to inform the hearer. Another way to express this point is to say that the speaker’s utterance must be not only intentional (this is clause 1), but overtly intentional (clause 2). The function of this second clause is to exclude behaviours that are intended to produce a particular response, but which are not meaningful. In the previous article I illustrated this distinction with the following contrast (derived and adapted from Grice 1989; Wharton 2009):

- Mary intends that her mother sees that she is unwell. Mary thus greets her mother with an exaggeratedly sad face, and overtly points to her forehead, which is pale.
- Mary intends that her mother sees that she is unwell. However, she doesn’t want this intention to be noticed (it might decrease her chances of getting a day off school). So Mary pretends to be asleep, but ensures that her pale forehead is fully visible.

Only (a) is overtly intentional. Hence, it is only (a) that can have meaning. Overt intentionality is not all that there is to meaning, but it is the heart of the matter (see citations above). Moore agrees: “this distinction [between (a) and (b)] captures Grice’s aims well” (in press, p. 3).

It is worth stating explicitly that, in this discussion to date, overt intentionality has effectively been used synonymously with the term ostension. For instance, my article was concerned exclusively with overt intentionality, but Moore interprets it in terms of ostension [“(Scott-Phillips’) argument turns on a distinction between gestures that are produced intentionally and gestures that are produced both ostensively and intentionally” (in press, p. 1)]. I will in this article continue to use the term overt intentionality, for two reasons. First, the label overt intentionality brings attention to the fact that plain intentionality—much studied in the animal communication literature—is insufficient for meaning. Overtness is necessary also. Second, the term ostension is not theory-neutral. As a piece of theoretical terminology, ostension was first used in *Relevance: Communication & Cognition* (Sperber and Wilson 1995) and continues to be closely associated with Relevance Theory. Still, Relevance Theory is only one of several theoretical approaches to linguistic pragmatics, and for the purposes of a short article I wanted to abstract away from those debates. Overt intentionality captures what is important about ostension and meaning, while remaining largely theory-neutral.

## How should we operationalise overt intentionality?

As mentioned above, there is agreement that evidence of a command over the difference between overt and covert intentionality would be good evidence of meaning in the relevant sense [i.e. (a) vs (b) in the example above]. Disagreement begins with the question of how this distinction could be experimentally demonstrated.

In my previous article, I suggested that experimental demonstration that individuals can differentially use overt and covert intentionality in appropriate contexts would be sufficient evidence for meaning. Above, Mary’s actions are overt in (a), covert in (b), and hence express her intentions in overt and covert ways. There is, to my knowledge, only one existing study of this sort, conducted on human children (Grosse et al. 2013). Three- and 5-year-olds played with an adult and in the course of doing so found themselves in a position where they had both a reason to help the adult, and the knowledge to do so. In the experimental condition they were also aware that the adult did not want to be helped. A control condition included no such restriction. In short, the children had a desire to inform the adult, but whether it would be appropriate to express this overtly (i.e. by normal means of Gricean communication) or covertly varied across conditions. In both age groups the children behaved according to condition: those in the control condition expressed their intentions overtly, but those in the experimental condition often did so covertly (Grosse et al. 2013). This type of covert behaviour is typically called *hidden authorship*.

Since they make extensive use of verbal interaction, these same methods cannot be used for non-human apes, or even for pre-linguistic children. These methods also rely upon children’s natural disposition to help others, which is not shared by other apes. As such, it is unlikely that these particular methods could be adapted for use with non-human great apes or pre-linguistic children. A new experimental protocol will have to be developed. Still, although there are serious methodological challenges here, it remains the case that the best, clearest evidence of overt intentionality is the ability to distinguish it from other forms of intentional expression, such as covert intentionality (see above). Nowhere does Moore dispute that success at such tests would provide compelling evidence of Gricean meaning. I assume this to be a point of agreement.

Moore does, however, offer some alternative suggestions about what might also constitute sufficient evidence for Gricean meaning. He focuses in particular on eye contact, which humans often use to express overt intentionality (this is not the only way in which they do so, of course). Moore points to several studies which manipulate whether or not eye contact is used in this way, and test whether participants (typically human children) behave accordingly. If participants are able to competently recognise Gricean communication for what it is, then they should interpret an individual’s behaviour as communicative only when overt intentionality is expressed. Several studies now show that human children of various ages are able to do this (e.g. Tomasello et al. 1997; Behne et al. 2005; Gräfenhain et al. 2009; Moore et al. 2013). Based on the success and general acceptance of this approach, Moore then suggests that eye contact can be used as diagnostic of the expression of overt intentionality. He further points out that eye contact often precedes ape gestural communication and hence concludes that there are sufficient grounds to assign meaning to this type of communication.

Here we reach a critical point. In the studies cited above, and others like them, eye contact is used to express overt intentionality. This does not mean, however, that the use of eye contact logically implies that overt intentionality is being expressed. It is quite possible to express overt intentionality without eye contact, and also for eye contact to occur without any overt intentionality. In fact, eye contact without overt intentionality is an entirely quotidian occurrence. It happens, in particular, if I simply want to check whether somebody is looking at me. For instance, when I am crossing the road, I sometimes make eye contact with a car driver, but do not express overt intentionality when I do so, at least not as a matter of course. In short, just because eye contact *can* be used to express overt intentionality, that does not mean that it always or necessarily *does* so. In short, eye contact is neither necessary nor sufficient evidence for overt intentionality. Moore is correct that there is “overwhelming” evidence for eye contact in chimpanzee communication, but this does not, in and of itself, imply meaning. There is overwhelming evidence that pedestrians use eye contact when they cross the road, but that does not mean that they express meaning when they do so.

The same points apply, *mutatis mutandis*, to infant communication, and also to other potential markers of overt intentionality, such as pointing. Just as the use of eye contact is not conclusive evidence of overt intentionality in any non-human species, neither is it in humans. Nor is pointing conclusive evidence either. Pointing, eye gaze, and some other behaviours can be and are used to express overt intentionality, and as such observation of such behaviour can thus be relevant to the discussion. A systematic and exhaustive comparison of expressive behaviour in infants and chimpanzees would be especially informative. But whatever such research might reveal, it will remain the case that eye gaze, pointing, and related behaviours are, formally, neither necessary nor sufficient for meaning.

The bottom line is this. To properly test for the expression of overt intentionality (and hence for meaning), then the key experimental tests are those in which overt intentionality is behaviourally distinguished from other means of intentional expression (such as covert intentionality), and where experimental manipulations predict which type of intentional expression should be employed. (To repeat: I believe that Moore would agree with this.) This is just as with any other cognitive process: we must behaviourally distinguish the process from other closely related behaviours and experimentally show that these different behaviours are performed exactly when we expect the different cognitive processes to be employed. With regard to overt intentionality, there is one such experiment on human infants (3- and 5-year-olds; Grosse et al. 2013), and none for any non-human animal, or any pre-verbal human infants. Moore’s arguments do not change these facts.

## What is the most appropriate working hypothesis?

Given that the key experiments for meaning in non-human animals have not been conducted, we cannot know for sure whether any non-human communication system has meaning, in the Gricean sense of the term. Ditto for pre-linguistic infants. This raises the question of what the right working hypotheses should be. In absence of definitive experimental results, we can therefore only (and arguably should) ask what is most likely to be true, based on present circumstantial data, and be willing to reassess as and when more conclusive data comes in. In short, we should be Bayesian about our working hypotheses. It may, moreover, be the case that the correct working hypothesis is different for children than for, say, chimpanzees (or any other non-human species). In this section I will suggest that this is indeed the case. Specifically, I will suggest that the circumstantial data are different for the two different groups, and therefore that we should assign different prior probabilities to the claims that (1) pre-linguistic infants communicate with Gricean meaning, and (2) non-human primates communicate with Gricean meaning. These prior probabilities can and indeed should change as further data comes in, but based on our present knowledge we should be more willing to believe that pre-linguistic infants are Gricean communicators, than to believe that non-human primates are.

In the case of pre-linguistic infants, it is highly relevant that they will in time develop into fully competent users of overtly intentional communication. Furthermore, newly linguistic children have been experimentally shown to have command of the distinction between overt and covert intentionality, and also of other important aspects of Gricean communication, in particular an intention to change mental states (rather than just behaviour) (Grosse et al. 2010, 2013; see also Tomasello 2008, for a review). Thus, to sustain a hypothesis that pre-linguistic infants are not Gricean communicators, we would be obliged to provide (1) an explanation of their pre-linguistic communication that is not Gricean, and (2) an account of how they transition from this non-Gricean state into a Gricean one. This may be possible, but the task is certainly not a trivial one. As such, the theoretical choice here is, roughly, between the following two views:

- that pre-linguistic infants are likely to be Gricean communicators from the beginning, and that what develops over time is their competence in this domain; or
- that pre-linguistic infants are not likely to be Gricean communicators, and hence that what develops over time is Gricean communication itself.

Both views are defensible, but there is no particular reason to assign them the same prior probability. As with all academic debates, there may be good reasons to believe that, absent definitive evidence, one view is, on balance, more likely to be true than the other.

With non-human primates the situation is at least somewhat different. Whereas experiments have shown that older children are competent Gricean communicators, there is no experimental demonstration of the same in non-human primates. In particular, there is no experimental evidence that non-human primates of any age or any degree of enculturation have command of the distinction between overt and covert intentionality, or that they have any intentions to affect mental states rather than behaviour. I am not arguing that such evidence will never be produced: maybe it will, maybe it won’t. What I am saying is that such evidence does not presently exist. We can explain this fact in (again speaking just roughly) one of two ways. Non-human primates have not been shown to demonstrate overt intentionality either:

- because non-human great apes most likely are Gricean communicators, but experimentalists have not yet been able to meet the significant methodological challenges required to demonstrate this; or
- because non-human primates are unlikely to be Gricean communicators.

Again, both views are defensible. Also as before, there is no particular reason to assign them the same prior probability. One or the other of these views may deserve a higher prior probability.

Note that the entailments of (1a) (that human infants are likely to be Gricean communicators) are not the same, *mutadis mutandis*, as the entailments of (2a) (that at least some non-human great apes are likely to be Gricean communicators). For instance, justification of (2a) requires some explanation of, among other things, the relative dearth of pointing in the wild. By raising the issue of pointing in the wild, I am not attempting to argue for one view or another. Rather, I am simply pointing out that this is a pertinent question (one of several), if we make a provisional acceptance of (2a). I am also pointing out that the list of pertinent questions for (2a) is not the same as those for (1a). Nor are the entailments of (1b) the same as those of (2b). Some key questions for each of the four views are listed in Table 1.

**Table 1:** Pressing questions for the views that non-human great apes, and pre-linguistic human infants, are likely/unlikely to be Gricean communicators

Group	Likely or unlikely to be Gricean communicators?	Open questions
Non-human great apes	Likely [proposal (2a) in the main text]	Why do non-human great apes not point for each other in the wild, and rarely in captivity? Why do unenculturated non-human great apes perform poorly at the object-choice task? How can the hypothesised ability to communicate in Gricean way be squared with the fact that, in comparison with human infants, non-human great apes have poor social cognitive skills? Given that they readily develop cultural traditions in other domains, why do non-human great apes not develop anything that could be called a language (i.e. a system of cultural traditions that enhances the expressivity of Gricean communication)?
	Unlikely [proposal (2b) in the main text]	Some cases of non-human great ape communication appear similar to some everyday cases of human Gricean communication. Are the two cases really so different?
Pre-linguistic human infants	Likely [proposal (1a) in the main text]	On many analyses, the social cognitive abilities required for Gricean communication are quite sophisticated. Do we really believe that pre-linguistic infants possess such abilities?
	Unlikely [proposal (1b) in the main text]	If it is not Gricean, then what sort of communication is this? How does it differ from Gricean communication? How and why do children transition from one type of communication to the other?

As such, there is no a priori reason that our choice between (1a) and (1b) should be equivalent to our choice between (2a) and (2b). Put another way, if we choose, say, (1a) over (1b), that does not mean that we should necessarily accept (2a) over (2b). The two choices to be made here are not the same as each other and each should be assessed on its own balance of probabilities. It would in particular not be anthropomorphic (at least not necessarily so) to accept (1a) over (1b), but (2b) over (2a). Indeed, my own reading of the present literature is, yes, that (1a) should be preferred over (1b), and that (2b) should be preferred over (2a). I have discussed my reasons for this belief elsewhere, and so will not regurgitate them here (see Scott-Phillips 2014, 2015a, b). Still, absent definitive experimental evidence (for discussion of which see above), individual researchers can form their own views about the balance of prior probabilities here.

## Conclusion

Questions about whether non-human primate and pre-linguistic human infants are Gricean communicators are presently matters of judgement, but the issues are in the end empirical matters. There are, moreover, key experimental tests that can be conducted, specifically experiments in which overt intentionality is behaviourally distinguished from other means of intentional expression, and where experimental manipulations predict which type of intentional expression should be employed. Only when such experiments are conducted will we be able to settle the matter. In the meantime, our claims can only be provisional. I have argued previously that the best provisional view for non-human primate communication is that it is most likely not meaningful in the same way that human words are, and Moore’s arguments (in particular regarding eye contact) do not persuade me to change this view. Regardless, let me finish by thanking Moore for his comments. Even though I disagree with the content of his arguments, I do believe that they make a worthwhile contribution to discussion. They have in particular forced me to sharpen my own views and arguments, and for this I am especially grateful.
